# Referral of Patients with Nonmalignant Chronic Diseases to Specialist Palliative Care: A Study in a Teaching Hospital in Ghana

**DOI:** 10.1155/2020/8432956

**Published:** 2020-03-15

**Authors:** Rasheed Ofosu-Poku, Michael Owusu-Ansah, John Antwi

**Affiliations:** ^1^Directorate of Family Medicine, Komfo Anokye Teaching Hospital, Kumasi, Ghana; ^2^Directorate of Internal Medicine, Komfo Anokye Teaching Hospital, Kumasi, Ghana

## Abstract

Ghana's chronic disease burden is on the rise. An essential aspect of clinical care in chronic disease management is to improve the quality of life of both patients and their families and to help them cope with the experience of life-limiting illness. Specialist palliative care services help reach this objective, especially in the context of complex psychosocial challenges and high symptom burden. It is, therefore, necessary that as many patients as possible get access to available specialist palliative care services. This paper explores the factors influencing referral of patients with nonmalignant chronic diseases for specialist palliative care. A qualitative approach was used to explore these factors from eight (8) participants—four (4) physician specialists and four (4) next of kin of patients with advanced nonmalignant chronic illness. Individual face-to-face interviews were conducted using a semistructured interview guide. Interviews were audio-recorded and data coded, themes and subthemes were identified, and thematic analysis was done. Barriers and motivators identified were categorized as either related to physicians, institution, or family. Barriers to referral were perception of the scope of palliative care, medical paternalism, lack of an institutional referral policy, poor human resource capacity of the palliative care team, and lack of awareness about the existence of specialist palliative care service. Poor economic status of the patient and family, poor prognosis, previous interaction with the palliative care team, and an appreciation of patients' expectations of the healthcare system were identified as motivators for referral. The palliative care team must therefore increase awareness among other health professionals about their services and facilitate the development and availability of a clear policy to guide and improve referrals.

## 1. Introduction

Chronic noncommunicable diseases (NCDs) such as cardiovascular diseases, cancer, and diabetes mellitus, account for 70% of all deaths globally [1], 75% occurring in the low- and middle-income countries (LMICs) such as Ghana [2, 3]. Morbidity and mortality from NCDs in these countries are projected to rise over the next decade, with five times as many deaths and three times disability-adjusted life years as communicable diseases by 2030 [4]. The leading causes of NCD-related deaths globally are chronic respiratory diseases, cardiovascular diseases, cancer, and diabetes mellitus, with cardiovascular diseases topping the list in Ghana [2, 5–9].

These chronic diseases have the inherent characteristic of being ominous—progressively advancing with worsening of symptoms, limiting quality of life and ability to function. Consequently, the physical, psychological, social, and spiritual facets of life of their victim are all affected remarkably [10]. To provide a holistic perspective to care, the World Health Organization recommends that palliative care be initiated along with life-prolonging therapy [11].

The World Health Organization defines palliative care as “an approach that improves the quality of life of patients and their families facing the problem associated with life-threatening illness, through the prevention and relief of suffering by means of early identification and impeccable assessment and treatment of pain and other problems, physical, psychosocial and spiritual” [11]. Thus, the overriding objective of palliative care is to enhance the quality of life of patients and their families by helping them meet their needs.

In order to integrate palliative care into the existing healthcare system, primary healthcare providers must start basic palliative care [12] but refer to specialist palliative care teams when symptoms are intractable, complex psychosocial issues arise [13], and/or the patient is likely to die within a year [14].

Palliative care development in the developed western countries such as the United States of America and the United Kingdom, as well as in East and South Africa, has advanced rapidly [15, 16]. However, its development and progress in West Africa has been rather slow [17]. In Ghana, it was not until 2003 that Ripples Health Care, a private firm, initiated a home-based palliative care service. Later, in 2006, the Ghana Palliative Care Association was formed [18].

Although it was in 2003 that Dr. Kwaku Afriyie, Ghana's then Minister of Health, raised the issue of the importance of incorporating palliative care in the nation's healthcare system, it was in 2012 that the national policy for control of NCDs highlighted the training of health professionals in palliative care in order to improve services for patients suffering from NCDs such as cancer, cardiovascular diseases, renal failure, and stroke [19, 20]. The National Cancer Control program has also devoted an entire chapter of the national strategy to palliative care, detailing how to get it properly integrated into the healthcare system towards management of patients suffering from cancer [21]. At present, specialist palliative care service in Ghana is provided by the Korle Bu Teaching Hospital (KBTH), the Tetteh Quarshie Memorial Hospital, and the Komfo Anokye Teaching Hospital (KATH).

Specialist palliative care services at KATH started in 2015, under the Family Medicine Directorate. Its models of care include in-patient consultation, out-patient clinic, and home visits. The hospital has no in-patient palliative care unit. Services provided include management of symptoms, discussing diagnosis and prognosis, negotiating goals of care, assistance with advanced care planning, end-of-life care, and bereavement support.

An essential requirement for the management of symptoms in patients with chronic diseases is the availability of syrup morphine for the management of moderate to severe pain. Morphine is imported as a powder and reconstituted into syrup by the hospital. Syrup morphine is therefore quite easily available within the hospital, albeit at a price equivalent to about 6 USD for a 100 ml bottle. Some pharmacies also have syrup morphine on sale, but at a higher price of about 15 USD for a 100 ml bottle. Apart from pharmacies in KATH, KBTH, and the country's two main cities (Kumasi and Accra), syrup morphine is hard to come by in other parts of Ghana.

Patients are seen based on referrals/consults from their respective primary physician. Most patients tend to be referred when their primary care team(s) appears to have run out of options in offering disease-modifying therapy, and patients have a few weeks to several months to live.

A review of the 2016 data on referrals to the hospital's palliative care team revealed that 49 out of fifty referrals (98%) were on account of an advanced cancer and only one (constituting 2%) was due to a nonmalignant chronic disease (end-stage renal failure). A similar observation has been made in the literature with respect to the pattern of referrals to specialist palliative care internationally [22]. This trend leaves out all those other patients suffering from nonmalignant NCDs such as heart failure, cerebrovascular accident, diabetes mellitus, and chronic obstructive pulmonary disease.

As the hospital is a tertiary facility, patients normally present with acute phases of chronic diseases, chronic diseases challenging to manage at the primary or secondary facilities, or acute severe disease. Patients at these stages of chronic disease generally require the support of a specialist palliative care team to help meet their complex needs [13, 14]. An appreciable number of consults/referrals each month for the palliative care team's input, to help address the needs of these patients, are therefore expected.

Referrals to specialist palliative care have been associated with many benefits to the patient, family, and hospital. These benefits include increased bed turnover rate, improved patient and family satisfaction with care, reduced hospital bills and associated costs, reduced number of emergency room visits, reduced caregiver burden, improved quality of life of patients, improved transition of care from high to lower acuity settings, improved pain and symptom management, and improved communication with patient and family about end-of-life issues such as advanced directives [23–32].

The hospital not only loses the above benefits but also has a high risk of incurring financial losses because patients may take unduly long periods in the ward but relatives may not be able to pay the accumulated hospital bill. Secondly, patients requiring acute care and stabilization in the ward have to spend days at the emergency department before a bed can be secured for them in the wards.

In facilitating the hospital's vision to become a centre of excellence in the provision of specialist care [33], this gap in access to specialist palliative care for patients with nonmalignant chronic diseases needs to be investigated and appropriate solutions identified and implemented as a matter of urgency.

Identification and comprehension of the factors influencing the decision to refer patients for specialist palliative care is necessary in planning appropriate interventions for implementation. However, in view of the fact that specialist palliative care in Ghana is less than a decade old [18], and barely half a decade at the Komfo Anokye Teaching Hospital, studies investigating these factors have not been carried out yet.

This study is therefore a need of the time in order to plan appropriately towards improving access to specialist palliative care, especially for patients with nonmalignant chronic diseases. This study explores these factors from the perspective of physician specialists and relatives of patients.

## 2. Materials and Methods

### 2.1. Study Design

A qualitative exploratory study design was used. This is because palliative care is quite new in the Ghanaian context, and there is very limited existing evidence base pertaining to the setting. Secondly, the individual perspectives of participants are the focus of this study [34].

### 2.2. Study Site and Population

This study took place at the Komfo Anokye Teaching Hospital, Kumasi, the second leading teaching hospital in Ghana. Specialist palliative care service is run by the palliative care team under the Family Medicine Directorate. According to the daily schedule of the palliative care team, it organizes both a home visit and an in-patient consultation twice a week and an out-patient clinic once a week. By the end of October 2018, the core team was made up of two family physicians, one surgeon, and two nurses. With the exception of one nurse who had been chosen by the hospital management to lead the formation of the palliative care unit, each of them had other primary duties and worked with the palliative care team if and when it had patients.

Another of the hospital's 12 clinical directorates is the Internal Medicine Directorate. Complicated diabetes mellitus and chronic neurological and cardiovascular diseases, which are the focus in this study, are managed by this directorate.

In teaching hospitals, physician specialists head the patient's primary care team, where they act as the “gatekeepers” of the referral process [35]. Physician specialists, therefore, constitute a major group in this study. Those included in the study had practiced for at least one year as a physician specialist in the Internal Medicine Directorate.

Secondly, patients and their families have the right to access or not palliative care service following discussion and referral by primary physicians [36]. As close relatives, the closest being the next of kin, are recognized to make decisions on a client's behalf in the event of incapacitation and even act as the main support for the patient during admission, their views about palliative care are important in the referral process. They are, therefore, included as the second group of participants in this study.

### 2.3. Sampling Technique and Size

Purposive sampling was used to recruit physician specialists and next of kin of patients who meet the inclusion criteria. The sample size consisted of eight (8) participants—four (4) physician specialists and four (4) next of kin, as determined by data saturation—“when no new analytical information arises anymore, and the study provides maximum information on the phenomenon” [37].

### 2.4. Data Collection Instrument and Method

Two (2) semistructured interview guides (one for physician specialists and the other for next of kin of patients) were developed and used to allow flexibility in exploring topics [38]. The interview guides were developed based on the objectives of the study, a review of relevant literature, and policy.

Each interview guide was reviewed by a supervisor to ensure that questions reflected the purpose of the study. They were then pretested with one participant each, a next of kin of a patient and a physician specialist. The purpose for this was threefold: to ensure the clarity of questions, to fine-tune the questions, and to provide an opportunity to practice and improve interviewing skills. The data collected during the pretest interview was not added to the study.

Following ethical approval, folders of patients on admission were reviewed to get to know patients with a diagnosis of a nonmalignant chronic disease with at least one complication. Those who were unconscious or could not communicate were excluded. A brief discussion was held with each of these selected patients to tell them about the study and to have a discussion with their next of kin. The next of kin were met in person during visiting hours, and participation in the study was discussed with them. Five out of eight consented and participated in the study.

To recruit physician specialists to participate, the principal investigator was present in the various wards of the directorate at various times of the day. Having worked in the directorate for a number of years, he knew those who fell within the inclusion criteria of the study and who among them could be most informative. Nine physician specialists were contacted in person and the study discussed with them. Of these, one declined participation on account of a busy schedule, four had busy schedules so there were challenges arranging a suitable time within the study period, and four participated in the study. These interviews were carried out in English.

Each participant was given an information leaflet and had it explained to him/her, and they each signed a consent form before their respective interviews. Individual face-to-face interviews were conducted in offices of various wards in the directorate. The purpose of employing individual face-to-face interviews was to encourage flexibility of time and venue for each participant and to encourage expression of diverse opinions [39]. Interviews lasted an average of fifteen (15) minutes, were conducted from 1st June, 2018, to 8th June, 2018, and audio-recorded with permission from the participants. At the end of each interview, the participant was thanked for their cooperation and offered no monetary reward.

### 2.5. Data Handling and Analysis

Interviews were transcribed verbatim and field notes added as needed. Transcripts were read by the supervisor to ensure rigor and trustworthiness. All names were replaced with pseudonyms. Signed consent forms have been kept safely in a locked cabinet and will be kept for at least five (5) years. Electronic data (transcripts and audio-recording of interviews) have been encrypted and password protected to ensure confidentiality of the study's participants.

Thematic analysis was used because of its flexibility, enabling its use in any theoretical or conceptual framework of choice, and as it allows for detailed description of data [40]. Transcripts were read and reread to gain familiarity with the data and identify the central concepts and key statements made by participants. Coding was done by assigning to sentences a phrase which conveys its purport. Codes were categorized into subthemes and subthemes into themes. Discussions were held with the supervisor to ensure that theoretical inferences are justified and personal opinions did not sway the analysis.

### 2.6. Methodological Rigor

Credibility was ensured through sessions with the thesis supervisor to get feedback about the quality of the data. Transferability was ensured by giving thick descriptions of the study setting and the context of the practice of specialist palliative care in the hospital. Dependability was ensured by working closely with the supervisor throughout the study and keeping an audit trail of all events and procedures followed. Detailed descriptions of the research design, data collection procedure and analysis, and basic information about study participants have been provided. Confirmability was ensured by working closely with supervisors who audited the data and inferences drawn from it. An audit trail of audio-records, transcripts, interview questions, and consent forms have also been kept for any future confirmatory audits.

### 2.7. Ethical Consideration

Ethical clearance was obtained from the Committee on Human Research Publication and Ethics (CHRPE) of the Kwame Nkrumah University of Science and Technology. Participants were each given an information leaflet and had it explained to them by the researcher. Those who accepted to participate completed a consent form.

In order to ensure the anonymity of respondents, they were not addressed by name during the interviews nor were their actual names used in the transcript of the interview. A code name was rather assigned to each of the participants in the transcript. Also, consent forms have been kept under lock and key, and interview transcripts and audio-records were encrypted and password protected. Participants were thanked for making time for the interview but were given no material reward as compensation.

## 3. Findings

### 3.1. Physician-Related Factors

The physician specialist interviewed had neurology and nephrology as their areas of specialization (Table 1).  Table 2 illustrates the diagnosis of patients whose next of kin were interviewed. Patients' diagnoses were related to cerebrovascular accident (stroke), liver disease, and diabetes mellitus type II.

Themes and subthemes emerging from the study have been illustrated in Table 3.

#### 3.1.1. Awareness of Specialist Palliative Care Services in the Hospital

Some physician specialists were neither aware of the hospital's ongoing specialist palliative care services nor aware of any palliative care physician specialist in the hospital. One participant said, “I don't know of anyone really in KATH who is a palliative care specialist or anything like that” (PS 4). Another physician specialist said, “We don't even have a place to send them to” (PS 2).

Another reason identified to be contributing to the level of awareness about specialist palliative care services was the lack of interaction between the palliative care team and physician specialists. One physician specialist said,

Well, in the first place I'm aware that there is a palliative care team at the polyclinic but we hardly have any interactions. So the first step is to recognize that yes there is a palliative care team and if you want to contact the palliative care team this is their contact number, this is who is in-charge, this is how we can contact you to become part of our team. (PS 3)

Previous experience with patients and the scope of interest of physician specialists were noted to be significant in finding out the availability of a particular service to meet certain needs of patients. In view of his previous study in the quality of life of patients, one physician specialist had identified a family physician with specialty interest in palliative care, who he contacted when he thought a patient may needed palliative care.

… I actually conducted a study on quality of life and indeed quality of life is something that now has become an important outcome measure for our patients with chronic kidney disease and I've done some studies even in quality of life and to tell you plainly it's so poor. And some of the things that came out from my study… we as physicians should not only be concerned about controlling blood pressures alone but we should think about patients in terms of their mental and psychological wellbeing because most of them come depressed. I mean you wake up one morning and you are told your kidney cannot function well and you need dialysis which is expensive…so I had a discussion with a colleague of mine who has interest in palliative that well I will be sending some cases for him to see because I get to points where they cannot afford dialysis, they are just lying on the ward and probably we are just waiting for them to die. (PS 1)

#### 3.1.2. Perception about Palliative Care

Physician specialists held different opinions about the most appropriate time for a palliative care referral. Whereas some thought palliative care should be started at diagnosis, others were of the view that palliative care may be started when the patient is unable to afford life-prolonging therapy or available treatment is not helping.

Physician specialists inclined to neurology were of the view that palliative care must start at the time of diagnosis. One of them said,

Look … when patients come with stroke and they are admitted, and doctors round on those patients you want a palliative care team to be part of that rounds who will be able to for instance meet with relatives and discuss prognosis in a very practical manner with them and how interventions that are palliative in nature can be instituted so that the patient even when the patient is going to die from the stroke at least the process is well managed in terms of the psychological and social implications. So from the word go, yes, palliative care team should be involved. (PS 3)

Another physician specialist said,

Umm, I think immediately the diagnosis is made. That is when. Because patients need to from the beginning be made aware what they are dealing with. They need to make life adjustments to deal with it for them and their family. They need to understand what they are dealing with. So I think that right from the beginning. (PS 2)

However, physician specialists with interest in nephrology pointed out that the triggers for a specialist palliative care referral are when prognosis is poor, the patient cannot afford available life-prolonging interventions, or available medical interventions are failing. One physician specialist was of the view that a specialist palliative care referral must be considered when the patient's condition is not improving with available medical interventions.

Of course, along the line there might be some other complications that may come on board, strokes and all that for patients with CKD, really bad electrolyte imbalances and all that which sometimes in spite of the dialysis… they do not do well eventually. So there are patients like that who in spite of the interventions will not do well. So may be for those patients, when it's looking like in spite of our intervention things are still ‘going south' then those people I think will benefit from palliative care. (PS 4)

He said once again, at the latter end of the interview,

… fine I'm in the renal clinic but we see a lot of other general cases, and for some of them its obvious things are not really going well… and they need palliative care. (PS 4).

Some physician specialists were also of the view that specialist palliative care consultation becomes necessary when a patient cannot afford the available life-prolonging therapy. That is, in their opinion, palliative care is incompatible with life-prolonging therapy and therefore cannot be considered when the patient is on interventions such as dialysis.

So I think most of them need financial assistance. And, because when you are put on dialysis and you are consistently on it for a long time I mean you can stay very long. So most patients there, I think what they need is not really palliative care but for those who cannot afford their interventions what they need is financial assistance… I think the main problem of treatment that the category of patients I have spoken about need is finances, because something can be done about their condition. For those who cannot get the things that can be done for them done, of course they can. It essentially comes down to finances. (PS 4)

Another physician specialist also said,

… now what I do is that, you know chronic kidney disease has stages so from stage 3 onwards to 5, that is when I start assessing for those I see early whether they can afford, yes and no, and how we can go around it. So I actually think sometimes depending on the background some people cope better than others. So sometimes you are talking with somebody, he is in stage 3, 4 but the coping ability is on the low side, then I think it makes sense to refer them early. (PS 1)

The perceived needs of patients and the roles their physicians perceived to be within the domain of specialist palliative care also determined the possibility of the involvement of specialist palliative professionals in the care of patients. To the physician specialists, the roles of specialist palliative care professionals include communication, patient and family support, symptom management, and advanced care planning.

One physician specialist, referring to the role of communication in palliative care, said,

What would have been ideal will be to have a multidisciplinary team, not only made up of neurologists but having nurses, physiotherapists, occupational therapist, nutritionist, psychologist and palliative specialist who will be able to manage patients when they come so that it's not just a matter of prescribing drugs but there is this aspect of social care where the relatives are involved and they are informed about potential outcomes of strokes and about how palliation can be administered for those who we know may not survive the stroke and even for those who are going to live with permanent disabilities some of which may involve some of them being in bed almost for the rest of their life. (PS 3)

Another physician specialist discussed his view of the role of specialist palliative care in supporting patients and family as follows:

Um definitely, because most of these things are not curative. They are things they have to live with for the end of their life. So they need a lot of support all through to the end. So, I mean, definitely, the way I understand palliative care,… allowing them to endure their life, making use of what they have till the end. And that is basically what they need in addition to rehabilitation. (PS 2)

Symptom management and advanced care planning were identified to be roles of palliative care professionals, and thus, needs, which when identified, may encourage physician specialists to refer patients for palliative care. One physician specialist said,

… so I had a discussion with a colleague of mine who has interest in palliative that well I will be sending some cases for him to because I get to points where they cannot afford dialysis, they are just lying on the ward and probably we are just waiting for them to die. So then in that case a bit of pain control here, a bit of how to make them comfortable, how probably to help them to write their last their wills. (PS 1)

A system in which healthcare professionals make available little information to patients and their family, and thus make all the decisions for them, make them only passive members of the healthcare team. This may result in delayed or nonreferral of patients who may otherwise benefit from specialist palliative care. One participant said,

I think it all depends on the doctor. If the doctor says you have to do something about it, why not. What do I know about it to say? Even if we have to bring him every week, I can put him in a car and bring him. So even if it's one or two days, when you finish seeing him, I can take him back. (NOK 3)

When asked if he may wish to suggest palliative care to the patient's doctor, one participant remarked,

For some of them if you say this to them, he may get angry. Or am I telling lies? ‘If you think I cannot take care of you then go.' And that will also cause you a problem. (NOK 2)

### 3.2. Institutional Factors

The unavailability of an institutional or working guideline, either of the hospital, department, or teams, was identified to be a major factor influencing palliative care referral practices. In response to the question of whether any protocol or guidelines were available to guide referral to specialist palliative care, one physician specialist said,

Not that I know of. And indeed for nephrology, I think I can speak more of, we do not have a clear protocol. Like I said, it's on as and when basis. So I think I assess my patients and I think this is what they need, so I just refer them for the appropriate, yeah. (PS 1)

Hinting at the unavailability of adequate human resource to meet the need for specialist palliative care, one physician specialist said,

It's [referring to specialist palliative care] a budding area so even if you want to be aggressive the human resource is not there, I mean. Hopefully, if you come out to help on the nursing side to help the colleagues who are physicians to be able to, to you know build it well. But as it stands, you know it's a new area and it's not every time they are around so we also have to bear with it. (PS 1)

One issue raised by a physician specialist was that various health professionals appear to be working in isolation with each guarding their respective “territories.”

…what is currently happening is we have a very disjointed post-stroke care where the patients come to neurology clinic and are seen and they have to go to physiotherapy for physiotherapy needs to be done and then if they have a nutritional care need they have to go and see a nutritionist. So it's disjointed. But if we have a comprehensive clinic where when a patient comes all these can be provided. Then that will be able to work. But people work in territories and people do not feel comfortable sometimes you encroaching on their territory, so that's one of the things that we need to overcome. (PS 3)

### 3.3. Family-Related Factors

#### 3.3.1. Awareness and Acceptability of Specialist Palliative Care Services

When asked whether they had ever heard of the expression “palliative care,” all next of kin interviewed responded in the negative. After explaining to them what palliative care is and the services provided by specialist palliative care professionals, they expressed acceptance of specialist palliative care services for their respective family members who were on admission. One respondent said,

Yes I will be interested and willing. You mentioned quite a number of services, about how we can take care of her. And I do not know those things I can do, so I think I should be able to see you so that you teach me so that the patient can also get better and I will also get better, I like it. (NOK 1)

#### 3.3.2. Family-Related Barriers

One concern raised was the possible cost of palliative care services. In response to whether he would engage or be interested in the services of palliative care professionals if the patient's primary physician discussed referral with him, one respondent said,

So do you render the service for free? I will be interested if I think I have the necessary support, made the needed preparation and can afford [touches pocket]. (NOK 4)

Distance from one's place of residence to the hospital was also identified as a potential barrier of access to specialist palliative care services. One respondent said,

What about if I am from far away? I hope you get what I mean? From what we are discussing, it is like the person is from Kumasi here, even if it is far it cannot be as far as Nkawie, Bibiani, Enchi. Enchi is the place where we came from. (NOK 2)

Another respondent said,

Where the house is, one has to walk let us say between here and the gate. So even if he is discharged, last time they were saying they will let him do physio, so when we are bringing him from the house, for me when he is discharged I have to leave and go back to work. So just look at this woman [patient's wife], can she carry this man over a distance of 200meters before coming to pick a car? It is impossible, it will not work. (NOK 3)

### 3.4. Suggestions

The development of protocols or guidelines on when to refer a patient for palliative care and what the role they will play in the management of the patient was suggested by physician specialists as a way of improving referral practices. One physician specialist said,

I think the first step will be for the neurology team and the palliative care team to sit down together and devise a protocol and then each one knows the contribution they are going to make to the patient and then putting those ideas together in setting up a protocol so that it's clear that okay this is what I can do for the patient. So at this point I will do A, B and C, and at this point you do AB, A and C. So I think that's the first step for the two teams to sit down together and devise a protocol or a guideline. (PS 2)

Another physician specialist said,

My suggestion is that I think we have to work together and they can also give us some input on how to go about it. You know then they let us know if you have a patient who fits a particular criteria A, B, C, D, okay let us have a look at this patient and we can advise. On the other hand, you have a patient who probably as per criteria is stable for you to manage then manage until this stage and you can refer to us. (PS 1)

Another suggestion given by physician specialists was that the palliative care team should get more vocal about their field by increasing awareness through presentations and interactions with colleagues from various departments. For instance, one physician specialist said,

I am a specialist I have been here for quite some time and I did not know so… I do not think people really know that you can get a consult for someone to be seen… like the way we do consults from other departments. So I think that's the first step. Or may be to make people aware because a lot of patients, fine I'm in the renal clinic but we see a lot of other general cases, and for some of them its obvious things are not really going well… and they need palliative care. So I think that's the first step. People should know that there is a palliative care specialist around. (PS 4)

Another physician specialist also said,

And maybe they should be a bit more vocal, they should come out, do presentations, lets know what it's really about… so my suggestion is they come out more, let other departments or people really know their importance so when the patient is getting to the stage of hospice and all, we know we have to call somebody. (PS 1)

One next of kin suggested that each ward must have a palliative care focal person who will identify patients who need specialist palliative care and obtain a referral for them.

So what I was thinking was that, you should discuss with them so that they get to know, so that for each ward one of your members can be there. (NOK 2)

## 4. Discussion of Findings

### 4.1. Physician-Related Factors

The study found that having knowledge about the existence of specialist palliative care services in an institution, knowing a colleague who has interest in palliative care, and previous clinical experience or interactions with specialist palliative care professionals were associated with increased likelihood of referrals from primary physicians to specialist palliative care professionals. This finding supports those of a multisite qualitative study in the United States in which lack of knowledge about the availability of palliative care services was a barrier to referral to specialist palliative care professionals [41]. A study by Opoku in 2014 among healthcare professionals and members of the general public in Ghana similarly found the unavailability of palliative care services as a barrier to provision of palliative care [17]. Also, in a study examining professional caregivers' experiences with palliative care for noncancer patients, they were found to have a “vague understanding of palliative care” and thus had difficulties identifying palliative care needs or initiating a conversation about palliative care [42]. In Nigeria, Onyeka and Iloanusi in 2014, describing their achievement in establishing palliative care in Enugu, have highlighted poor knowledge of palliative care among physicians as a contributory factor to low referrals previously recorded [43].

Another factor emerging from interaction with physician specialists, which had a direct effect on the possibility of the referral of patients to specialist palliative care, was their perception of the concept of palliative care. The findings centered on the appropriate time for a consult to specialist palliative care and the role of specialist palliative care professionals in the care of patients.

Physician specialists with interest in neurology, the majority of whose patients are suffering from stroke, pointed out the most appropriate time for involvement of the specialist palliative care team to be immediately when the diagnosis is made. However, those with interest in nephrology were of the view that specialist palliative care referral becomes necessary when prognosis is poor in spite of disease-modifying therapy being delivered or when the patient/family is in financial difficulties and hence cannot afford such therapy as dialysis. Although their responses seem to vary, one common issue is that acute stroke is life limiting/threatening, and like advanced chronic kidney disease, it may have a poor prognosis. Thus, both groups were of the view that poor prognosis was a trigger for specialist palliative care referral. This finding reflects the study among professionals caring for heart failure patients in which some health professionals believed that the right time to involve specialist palliative care is when disease-modifying therapy stops and prognosis is poor [44]. Similarly, in a study describing factors which predict the referral of cancer patients for specialist palliative care in Uganda, poor performance state (ECOG (Eastern Cooperation Oncology Group) 3 or 4)—a prognostic measure on the care of cancer patients—was the “only significant predictor of referral” [45].

The need to control physical symptoms, discuss diagnosis and prognosis with the patient and the family, provide them support, help them reintegrate into society following illness, and help them plan and manage affairs in the event of death were identified as reasons why physician specialists will contemplate the involvement of specialist palliative care professionals. This finding supports the findings of the study by Kavalieratos et al. [44] in which most healthcare providers based referral on reasons such as prognosis, symptom presence, care coordination, and advanced care planning. The study in Ghana exploring factors that affect provision of palliative care similarly found that the need for symptom management and holistic support were major reasons for initiating palliative care [17].

The above finding that patient's needs may trigger referral brings into perspective the fact that where a primary care provider feels confident in addressing an identified need, the patient is less likely to be referred for specialist palliative care. This inference is supported by the findings of a study among hematologists in which their confidence and comfort in addressing certain identified needs significantly influenced referral to specialist palliative care professionals [46]. Another study conducted by Low et al. [45] describing the referral pattern to specialist palliative care in Uganda further supports this inference. Their study showed that most physicians felt confident in dealing with a wide array of issues related to palliative care such as symptom management and basic communication and so referred patients for specialist palliative care when other needs of patients related to end-of-life care arose for which they were less confident. Thus, patients referred for specialist palliative care in their study were referred quite late, with a median survival of 5 days postreferral recorded [45].

Another factor identified in this study was that various professionals tend to work in isolation, “guarding their territories.” That is, some physicians do not permit other physicians to see their patients for various reasons, for instance, to prevent contradictory information from being communicated to patients and their relatives. A similar observation was made in a previous study in which some primary physicians avoided referral of their patients to specialist palliative care out of fear that palliative care professionals may present a “dismal” prognostic picture to patients and thus affect their willingness to continue to receive disease-modifying therapy [31]. Low et al. in 2018 similarly found in their survey that about 60% of doctors felt patients and family are more likely to lose hope and get depressed when end-of-life issues are discussed [45].

This study also found that close relatives of patients had left the entire burden of decision making about the plan of care for their loved ones on the primary physician. This reflects a feeling of inadequacy on the part of a patient's family about the progress of disease and options available. Such a state is indicative of the extent to which relations of patients are not abreast with care and are not deemed important in making decisions about the care of the patients. Following a review of several studies, Devlin and Maida in 2017 have highlighted the threat medical paternalism poses to specialist palliative care referral [47]. In addition, Adwedaa in 2015 had discussed how this practice of keeping information and making decisions on behalf of the patient and the family is common among doctors in Ghana [48]. It appears that doctors in Ghana keep information away from patients and their family in order to prevent them from losing hope, or they believe they know what is in the best interest of the patient and the family.

### 4.2. Institutional Factors

Another contributory factor to low referrals of patients for specialist palliative care is the lack of institutional or departmental policies and guidelines. Physician specialists were unanimous on the point that there were no guidelines for referral to specialist palliative care and recommended that clear guidelines are essential in ensuring access to specialist palliative care for their patients. This finding corroborates a study in Australia in which the availability of a team-based policy was a major motivator for referral to specialist palliative care [49]. In a recent study in Uganda in which the median period of survival after referral to specialist palliative care was found to be 5 days, the lack of a clear referral protocol was found to be a major contributory factor to the poor referral pattern [45].

This study also found that the unavailability of the required human resource capacity to meet the specialist palliative care needs of patients in the hospital may affect referrals. A study in Korea exploring doctor's perception and referral barriers towards palliative care similarly found the unavailability of resources for specialist palliative care such as nurses and doctors with relevant training as the second most commonly reported barrier [50]. Likewise, in Low et al.'s study in Uganda, referring doctors expressed concerns about the lack of adequate staff working with the specialist palliative care team and felt the team's poor staff strength cannot match the demand if referrals were to be made for all patients who need specialist palliative care [45].

### 4.3. Family-Related Factors

One finding emerging from this study is the unawareness of the concept of palliative care by relatives of patients. When patients or their close relations have no idea of a healthcare service, it is unlikely that they may raise a discussion about it during their interaction with their primary physicians. Especially in the context of patients suffering from nonmalignant chronic diseases, the use of the expressions like “palliation” or “palliative care” by health professionals is less common as compared to those with malignancies (cancers). It is, therefore, not abnormal for relatives of patients suffering from nonmalignant chronic diseases to have never heard the expression “palliative care.” This finding reinforces a nationwide survey of hospice and palliative care programs in the United States in which lack of awareness about palliative care by close relatives was rated as a major barrier to palliative care referral for noncancer patients [51].

Another concern expressed by close relatives of patients is the potential cost of services and the distance from their place of residence to the hospital and the stress involved. This may cause patients and their relatives not to turn in a referral to specialist palliative care. A study in a predominantly rural area in Norway similarly found that patients with lower socioeconomic status were unable to have follow-up sessions of palliative radiotherapy on account of the cost implications for treatment and transportation to the facility [52]. Another study exploring barriers to palliative care in Ghana also found the inability to pay for palliative care services as hampering the provision of specialist palliative care services [17].

Relatives of patients were receptive of the concept of palliative care after it had been explained to them and were interested in having palliative care professionals care for and guide them in the care of their loved ones. A qualitative grounded theory on the experiences of patients and caregivers with palliative care, in which participants expressed feeling a sense of holistic support, supports this finding [53]. Further corroborating the above finding of this study is a review of the palliative care service received by adult cancer patients receiving home-based care in Ibadan. Although patients had died at the time the study was conducted, relatives expressed profound gratitude to the palliative care team for their care and support [54]. However, the study by Lee et al. in 2012 reported the refusal of patients and their relatives as a significant barrier to specialist palliative care [50].

## 5. Conclusion

This study indicates that close relatives of patients are receptive of the concept of palliative care and are interested in benefiting from their services. However, they are generally unaware of its existence and are unable to initiate the discussion with their physicians because they think the physician knows best or may get angry and neglect the patient. As advocates for the welfare of the patient and relatives, nurses must assess patients and relatives and discuss involvement of specialist palliative care professionals with their respective physicians when needed. This, therefore, requires that not only should nurses be able to carry out a holistic assessment of patient and family, but that they should also be knowledgeable about palliative care and the triggers for specialist palliative care referral.

This study also revealed that close relatives of patients anticipate challenges with distance between their place of residence and the hospital, making the task of taking a patient to the hospital for review quite challenging. Another potential barrier to specialist palliative care revealed by relatives of patients was their financial constraints. The Ministry of Health must, therefore, formulate appropriate policies to ensure that training of specialist palliative care professionals is ongoing and that they are fairly distributed throughout the country for equitable access. To reduce the financial constraints on patients and families requiring specialist palliative care, the Ministry of Health must work towards ensuring that specialist palliative care consultation and essential medications used in palliative care are covered by the National Health Insurance Scheme.

Another major barrier is unawareness of available specialist palliative care services by physician specialists. The palliative care team must therefore carry out sensitization and interactive seminars with physicians, nurses, and other health professionals in the hospital to tell them about the services offered by the team and how they can be reached.

The unavailability of clear referral guidelines to specialist palliative care is also a major barrier. The hospital must therefore devote resources to the formulation of referral guidelines from various specialty areas to specialist palliative care.

This study focuses on physician specialists and close relatives of patients only, using a qualitative approach. It cannot be denied that nurses and the patients themselves are important members of the healthcare team, and the importance of their perspective cannot be overemphasized. Further studies could also explore these factors from their perspective using both a qualitative approach for an in-depth understanding of the factors and a quantitative approach to determine its scope.

Secondly, this study focuses only on the Internal Medicine Specialty. However, it is likely that not all patients suffering from cancer who require a referral to specialist palliative care receive it. So it is that, based on the peculiar needs of patients in other directorates such as Child Health, Emergency Medicine, Surgery, and Obstetrics and Gynaecology, some patients may require specialist palliative care but do not get referred. Further studies can look into these areas as well so that the majority of patients have their physical and psychosocial needs addressed through a healthcare system whose structures are evidence-based and proactive.

## Figures and Tables

**Table 1:** Physician Specialists and Their Areas of Specialization.

No.	Name	Specialty area
1	PS^∗^ 1	Internal medicine (with subspecialty interest in nephrology)
2	PS 2	Internal medicine (with subspecialty interest in neurology)
3	PS 3	Neurology
4	PS 4	Nephrology

^∗^PS is a codename used for physician specialists who participated in this study.

**Table 2:** Next of Kin and Diagnosis of Patients.

No.	Name of next of kin	Diagnosis of patient
1	NOK^∗^ 1	Infarctive stroke with right hemiparesis, type II diabetes mellitus
2	NOK 2	Acute-on-chronic liver disease with stage I encephalopathy, hypertension
3	NOK 3	Cerebrovascular accident
4	NOK 4	Cerebrovascular accident

^∗^NOK is a codename used for next of kin who participated in this study.

**Table 3:** Themes and Subthemes from Transcribed Data.

Themes		Subthemes
1	Physician related	(i) Awareness (ii) Perception about palliative care (iii) Medical paternalism (iv) “Territorial guarding”

2	Institutional factors	(i) Policy (ii) Human resource

3	Family related	(i) Awareness and acceptability (ii) Economic soundness

## Data Availability

Interview transcripts used for this study are available from corresponding author upon request.

## Additional Points

*Study Limitations*. As diabetes mellitus and cardiovascular diseases are among those nonmalignant chronic diseases which form part of this study's focus, we wished to interview physician specialists with interest in endocrinology and cardiology as well. However, on account of their busy schedule and the limited time available for data collection, they could not be included.
